# Applying global standards to short-term global health clinical experiences: the case of Project Salud y Paz

**DOI:** 10.1186/s12992-018-0445-7

**Published:** 2019-01-09

**Authors:** Paul H. Caldron

**Affiliations:** 1 Midwestern University Arizona College of Osteopathic Medicine, Glendale, AZ USA; 2 Phoenix, USA

**Keywords:** Short term, Global health, Position statement

## Abstract

This case study was extracted from an administrative report generated for Project Salud y Paz (SYP), a non-governmental organization (NGO) that supports short term global health clinical experiences (STGHCE) in Guatemala. As a basis to generate criticisms and offer recommendations, the analysis used the shared themes of two sets of standards recently published by Globalization and Health (GH) and the American College of Physicians (ACP). These standards respectively address the ethical responsibilities of organizations and of physicians in the conduct of STGHCE. Information used in the original quality analysis and quality improvement consultation for SYP was gathered from interviews, medical committee minutes, output statistics, and observations in the course of a real-time trip. This case study describes how the standards served as a useful lens to assess SYP and as a platform from which to make recommendations for improved compliance with global conventions. Additionally, the standards provide SYP a body of consensus wisdom for holding itself accountable to patients, host communities, volunteers, and the donor community on a continuing basis. While the shared themes of these standards are intentionally broad and require context in their application, NGOs that support STGHCE may find it instructive to benchmark them to assure their own compliance with global standards for both the organization and their volunteer physicians.

## Background

In the first quarter of 2018, Lasker et al published in Globalization and Health (GH) an overarching set of six core principles distilled from an analysis of 27 published sets of non-specialty-related guidelines aggregated in a scoping review [1]. Contemporaneously, the Annals of Internal Medicine (AIM) published an American College of Physicians (ACP) position statement derived by consensus of an expert panel regarding ethical obligations of physicians participating in short term global health clinical experiences (STGHCE) [2]. Broadly speaking, the GH principles are more relevant to the behavior of non-governmental organizations (NGOs) that conduct and support STGHCE while the ACP positions relate to physician conduct. Shared themes can readily be seen between the positions and principles of these two benchmarks (Table 1). ACP position 3, i.e., “The ethical principle of respect for persons, including being sensitive to and respectful of cultural differences”, may be viewed as integral in part to 4 of the 6 themes of the GH principles.

**Table 1:** Alignment of Globalization and Health Principles and American College of Physicians Positions

Standards and Shared Themes	
*Globalization and Health* [1] *Geared towards organizational behavior*	*American College of Physicians* [2] *Geared towards physician behavior*
1. Appropriate recruitment, preparation and supervision of volunteers	**Position 3.** The ethical principle of respect for persons, including being sensitive to and respectful of cultural differences, is essential to short-term global medical experience. **Position 4**. Pre-departure preparation is itself an ethical obligation. It should incorporate preparation for logistical and ethical aspects of STEGHs, including the potential for ethical challenges and moral distress.
2. A host partner that defines the program, including the needs to be addressed and the role of the host community in directing and teaching the volunteers	**Position 2.** The ethical principle of justice requires partnering with local leaders to ensure that the potential burdens participants can place on local communities abroad are minimized and preparing for limited material resources. **Position 3.** The ethical principle of respect for persons, including being sensitive to and respectful of cultural differences, is essential to short-term global medical experiences.
3. Sustainability and continuity of programs	**Position 5.** Physicians should participate with organizations whose STEGHs are consistent with ethics and professionalism as exemplified in these Positions.
4. Respect for governance and legal and ethical standards	**Position 3.** The ethical principle of respect for persons, including being sensitive to and respectful of cultural differences, is essential to short-term global medical experiences.
5. Regular evaluation of programs for impact	**Position 1**. Physicians’ primary ethical obligation in short-term global health experiences is to improve the health and well-being of the individuals and communities they visit.
6. Mutuality of learning and respect for local health professionals	**Position 3**. The ethical principle of respect for persons, including being sensitive to and respectful of cultural differences, is essential to short-term global medical experiences.

How these two sets will be utilized to affect the conduct of STGHCE remains to be seen. This case study provides an early example of the utilization of these positions and principles, herein referred to collectively as “standards” to benchmark the behavior of a specific NGO, Project Salud y Paz (SYP) operating in Guatemala. Herein, the analysis uses the standards in the GH order as cross-correlated in Table 1.

The millennium has witnessed a surge in health related volunteerism principally from the Global North to Global South [3]. These short excursions occur in a myriad of formats, from individual doctors to large faith-based and secular NGOs, in teaching and non-teaching domains, utilizing any number of social networks. Latin America draws this effort primarily from the US due to proximity effects, while Canada divides similar effort between Latin America and, stemming from its cultural relationship with Great Britain, the African subcontinent [4–6].The humanitarian zeal that drives the effort now draws substantial criticism in academic literature for actual and potential harms when executed poorly. The larger harms are perceived to arise from inadequate continuity of follow-up care, displacement of efforts by local clinicians and health administration, exceeding their scope of competence by visiting clinicians, exploiting the host country poor for learning purposes, and stressing local resources [7].

Although examples can be found of individual physicians from the Global North and South working together to provide direct care to the poor, most physician-volunteers work through established NGOs for their humanitarian expression.

The research aim in this exploratory, single case study report is to assess the facility with which these consensus standards can be applied in a real-life context and lead to meaningful recommendations.

Portions of the original administrative consult provided to SYP have been modified or rearranged to improve clarity in its utilization for this case report.

### Project Salud y Paz

SYP is a secular (NGO) operating in the western highlands of Guatemala. Its key focus is to increase the availability of health care primarily to the indigenous population at the lowest socioeconomic strata in Guatemala. The population served is largely of Mayan ethnicity whose economy is agriculturally based, as well as some artisan-related production. SYP currently has four full-time expatriate staff members from the US. SYP is the host-country operating arm of International Hands-in-Service, a non-profit (501c3) in the US which handles fund-raising as well as the purchase and exportation of pharmaceuticals utilized by the fixed clinics and the visiting teams. A comparable legal registry is maintained in Guatemala.

SYP has conducted missions from several faith-based (various Christian denominations, and Jewish) and secular traveling teams including medical, dental, and surgical teams. In the past 3 years, an estimated 30 teams per year have been supported from approximately 28 different clients.

In executing its medical service mission, SYP has established two full-time fixed clinics operating with sliding scale revenue in the communities of Camanchaj, department of El Quiché, and Nahuala, department of Sololá. It also maintains a part-time clinic in the town of Cunen, department of El Quiché, operating a fixed two days each month. These three clinics serve as hubs to spokes of 28 communities in the region where SYP confines its activities. The spoke communities are serviced by visiting teams of expatriate doctors largely from the US, Canada, and occasionally the United Kingdom, generally accompanied by one of the contracted Guatemalan physicians who man the fixed clinics when no traveling medical teams from abroad are in country. Such teams will generally visit one village each day of a one-week service trip to screen for acute and chronic medical illnesses, maintain continuity of care for chronic illnesses such as hypertension and diabetes, and make referrals to available secondary and specialist care within the national health care system of Guatemala. For follow up and ongoing care, these teams all direct patients to the fixed clinics. Lastly, individual villagers are identified as “health promoters” who may assist with monitoring patients in follow up for acute and chronic illnesses and facilitating follow-up in the fixed clinics. This avenue of support called “Las Amigas”, however, is largely inactive at this time.

A reflection of current productivity is indicated by raw aggregated figures that nearly 1000 visits at the three hub clinics were conducted during the first quarter of 2018 by the two contracted physicians. Most common illnesses addressed included type 2 diabetes, hypertension, upper gastrointestinal complaints, acute diarrhea, abdominal pain, degenerative arthritis, headache, respiratory infections, dyslipidemia, and dermatitis. Paradise Valley United Methodist Church (PVUMC) is an independent faith-based organization with a history of sending such teams to Guatemala annually since year 2000. On a typical five-village excursion in March 2018, the PVUMC traveling team from Arizona, USA with three volunteer physicians and one contracted physician conducted nearly 600 patient encounters in the week. Mechanisms for assessing individual patient progress or community impact remain undeveloped.

SYP leases its properties for the fixed clinics from the National Methodist Church in Guatemala (NMCG). Though separate entities, SYP maintains a collaborative dialogue with NMCG so that their individual objectives for the poor in this region are generally aligned.

There are three key sources of revenue to SYP. The first is donations aggregated through International Hands-In-Service from US churches and individuals. Second, margins are assessed on the package of services that SYP provides to volunteer client traveling teams. The package includes the arrangement of ground transportation, hotels, meals, insurance and the sale of T-shirts, coffee and some other local items specific to each team. Air transportation, insurance and team-specific T-shirts are arranged collectively by each team and are not included in the package of services provided by SYP. Lastly, SYP enjoys a margin on the medications provided in prearranged boxes for the teams, amplified somewhat by the return of unused products to SYP without refund at the end of a volunteer team excursion. Not quantified is the equity of staff employment that is salaried or stipend-supported below market value. A varying amount of additional income accrues from sliding scale charges at the hub clinics, generally representing 5-15 percent of total revenue.

The administrative consultation was arranged through personal networking of SYP administration and the author, instigated by the publication of GH and ACP standards in early 2018, to assess the organization’s compliance. The approach was 3-pronged, utilizing traditional business analysis methods: (a) a SWOT-based (Strengths, weaknesses, Opportunities, and Threats) semi-structured analysis questionnaire [8]; (b) 360-degree (multi-source/multi-rater) feedback, garnering input from available stakeholders, print and other resources (SYP was the focus rather than individual managers) [9]; and (c) the published standards were used as the enquiry benchmarks rather than other results, attributes, or compliance with mission statement.

Information used in the original pro-bono, third-party, consultative, quality analysis and quality improvement observation was generated from multiple sources. Interviews were conducted with the Executive Director, the Director of Informatics and Pharmacy Management, two current fulltime, contracted, Guatemalan physicians (hereinafter referred to as the “contracted physicians”), traveling team coordinators from Guatemala and from the US, and an administrative representative from the office of the president of the NMCG. Documentation resources included minutes of the medical committee, and representative output statistics. Interactions and observations within the course of a real-time trip with a client medical team from PVUMC provided additional insights. Juxtaposing the actions of SYP and PVUMC allows for a clarification of roles within the partnership. The relationship between SYP and its client organizations that send traveling medical teams to Guatemala injects a level of complexity to the analysis. Such complexity may be expected when organizations engage across borders and cultures in pursuit of productive ventures. The co-dependence of organizations in executing their core objectives renders an attempt to assess them independently artificial and less relevant. Lastly, more than 180 recipients of care including several community leaders who presented as recipients of care, though not interviewed specifically for their views on SYP, were directly engaged in the course of their care with the traveling team.

### Standards adherence and recommendations for compliance

The administrative report to SYP was organized according to themes. Table 2 displays the key recommendations derived from observations related to each of the shared themes synthesized from the GH and ACP standards.

**Table 2:** Key recommendations for Project Salud y Paz from GH and ACP standards assessment

Shared themes and recommendations	
Preparation and supervision (GH principle 1 / ACP Positions 3 and 4)	• The responsibilities for specific components of orientation between client NGOs and SYP should be spelled out so as to be comprehensive and complementary.
Host partner that defines the program and ensures that the burdens on local resources are minimized (GH Principle 2 /ACP Positions 2 and 3)	• Affirm host-country representation on the board. • Maintain the one-week limitation for traveling teams.
Sustainable and continuous programs that lend themselves to professionalism and ethical conduct (GH Principle 3 / ACP Position 5)	• Consult village leadership to identify appropriate candidates for the role of village advocates. • Deconstruct the traveling team timing trap. • Increase the number of visiting surgical teams in collaborative efforts.
Respect for local governance, standards and ethics (GH Principle 4 / ACP Position 3)	• SYP should seek written guidance from the MoH on the legality of US physicians administering care to Guatemalan citizens. • The Guatemalan medical director should collect and vet the credentials of all traveling licensed personnel prior to travel.
Regular evaluation of programs for impact specifically focused on healthcare (GH Principle 5 /ACP Position 1)	• SYP should court medical, surgical, and dental team sources that lack a host partner that is as well-organized. • Explore technologies that expand capacity and quality economically. • Maintain review and action loop of the board and medical committee. • Seek guidance through CUGH on strategy for capacity building before taking on an educational role.
Mutuality of learning and respect for local health professionals and their essential contributions (GH Principle 6 / ACP Position 3)	• Traveling and contracted physicians should pre-brief together on the eve of travel to review expectations and strategies in medical encounters.

#### Preparation and supervision (GH principle 1 / ACP positions 3 and 4)

##### Observations

Safety orientation from SYP to traveling team members is repeated throughout the week including precautions against consuming street food, unsealed beverage sources, and produce outside of reputable restaurant establishments, the hazards of street dogs and other animals, keeping in pairs or groups for excursions away from team activities, staying hydrated, hand washing, disposition of toilet tissue, skin and eye protection. Traveling members of the current PVUMC team indicated that pre-departure orientation included much of the same information. Some description of the indigenous Mayan population and culture, climate and geography are provided.

Preparation of new volunteers for psychological reactions they may experience on first confronting grinding poverty and the emotional reconciliations on re-entry to their usual lives was not evident from SYP. The need for overt discussions on the potential for culture shock may be substantially mitigated within the PVUMC group by its inclusion of three globally experienced physicians and several repeat support staff, the nature of the faith congregation, opportunities for reflection on the work in a spiritual context, and scheduled group prayer moments.

##### Recommendations

SYP and its client traveling teams should take steps separately or in concert to assure that each volunteer has been briefed and debriefed in this regard and offered resources for counseling with the experienced team members or other professional resources. Cultural orientation of all volunteers regarding local traditions and the comportment of guests within indigenous communities and among native staff should be more overt. Physical contact etiquette such as shaking hands, embracing, head-touching, hand symbols, patriarchal decision-making for the family, understanding the blend of Western religion and the elements of local belief structures should be reviewed to assure that all who interact with host citizens have the tools necessary to display adequate respect. Conversation about ideas and practices that prevail locally in contrast to what is better or worse, right or wrong, progressive or backward should have a place in the orientation process. In sum, the responsibilities for specific components of orientation between client NGOs and SYP should be spelled out so as to be comprehensive and complementary.

#### Host partner that defines the program and ensures that the burdens on local resources are minimized (GH principle 2 / ACP positions 2 and 3)

##### Observations

With its bi-national registration, SYP functions as the local host partner for outreach efforts of other secular and faith-based NGOs. The executive director, while consistently a US expatriate, appears to be bound in the mandate of assuring that organizational activities resonate with the views of host-country stakeholders, specifically native physicians and staff, village leadership, and partnering entities such as the NMCG. Other than the conduit of the executive director, there is no direct Guatemalan representation to the SYP board of directors. The Guatemalan medical director, in contradistinction, is an actively participating member of the medical committee, attending meetings real time virtually, and has submitted evaluation and treatment protocols for adoption.

The Guatemalan medical director expressed satisfaction with the duration of each visiting team’s journey, i.e., one working week, wherein 5 villages are visited. He cites two reasons for this: 1) the pattern strikes the right balance between the village outreach with traveling and contracted physicians working together, while not drawing the contracted physician away from the hub clinic for too long a period; 2) the effects on the traveling physicians of two weeks or more might be too strenuous and discouraging. Each day consists of hard bus travel on difficult roads and terrain, long hours of interviewing and examining patients, and evenings of preparation for the subsequent day. Lasker’s research in Africa suggests that host-country organizations prefer stays of two weeks or more from traveling volunteers [7].

##### Recommendations

The compliance of SYP with the relevant published standards could be underscored by affirming host-country representation on the board.

For SYP, striking the balance between fixed clinic and traveling for permanent physicians reflects the effort to avoid overburdening local resources in deference to the needs and requests of traveling teams. It is reasonable for the present to maintain the one-week limitation for traveling teams.

#### Sustainable and continuous programs that lend themselves to professionalism and ethical conduct (GH principle 3 / ACP position 5)

##### Observations

The output tracking of patient encounters, the establishment of a functional medical record system, and facilitation of traveling teams from various secular and faith-based outreach communities underscore SYP’s primary objective of addressing health inequality. Development of the hub and spokes service model with integration of the contract physicians and traveling teams has created a platform for incremental quality improvement. Exploiting the available skills to revolutionize pharmacy inventory, customizing team service packages, and using cellular technology systematically to facilitate continuity of care enhance the model’s efficiencies.

The conceptualization of village health advocates, Las Amigas, should be enhanced by these advances and reciprocally enable continuity of care and the implementation of future supportive technologies. Implementation of the initiative has languished from lack of a consensus strategy. Selection of appropriate village representatives is delicate, since too much training might perversely lead to the exodus of the trainee from their respective village.

A major challenge to SYP’s hub and spokes service model is that travelling teams most commonly want to schedule in coordination with the US spring, fall, and summer academic breaks.

##### Recommendations

Close consultation with village senior leadership could help identify individuals with strong ties as well as the necessary aptitude for a role in las Amigas village health advocates.

Strategies to escape the timing trap for traveling teams should be vigorously explored to optimize continuity. Therein may lay a key to stabilizing revenues to SYP. Increasing the number of surgical visiting teams collaborating with local surgeons could build both services capacity and the economic security of the organization.

The current lean budget threatens the scope of the mission. Unapologetically maintaining an adequate margin from operations to fund local staff and contracted physicians is paramount to the sustainability and impact of the long-term effort.

#### Respect for local governance, standards and ethics (GH principle 4 / ACP position 3)

##### Observations

The recent restoration of favorable relations with NMCG after serious disruption in the setting of diverging views between NMCG leadership and a prior SYP executive director reflects well on the current store of respect within SYP for local influence. The use of indigenous attire by the current executive director speaks volumes.

Dependence on contracted Guatemalan physicians ostensibly safeguards against tangential behavior and practice by traveling clinicians.

Vetting of professional credentials for physicians from the US is not actively pursued by either SYP or PVUMC. Traveling physicians have carried documentation of their home licensure, but generally have not been asked to produce such credentials. The webpage of the Guatemalan Ministry of Health (MoH) indicates only that health professionals should register confirmation of training with the MoH; no alternative regulation for visiting physicians is provided. It is the understanding of the executive director that permission from the MoH for short-term medical and surgical teams to function in Guatemala is not required. The contracted physicians affirm the absence of any requirements of registration for expatriate clinicians. It is noteworthy that, upon request, SYP has repeatedly obtained an invitation from the public health clinic in the district of Chichicastenango for specialty teams because the donor companies of eye surgery supplies require it.

##### Recommendations

SYP should seek written guidance from the MoH on the legality of US physicians administering care to Guatemalan citizens, notwithstanding concerns of official corruption. Until the matter can be reasonably clarified and documented, all physicians, advanced practice clinicians, and other licensed health care providers from the US administering to patients should, at a minimum, be required to provide documentation of current US (or other sovereignty) licensure to SYP and administer services only in the accompaniment of equivalently licensed professionals in Guatemala. The Guatemalan medical director of SYP should review and approve licensure documentation in advance of travel by US professionals.

#### Regular evaluation of programs for impact specifically focused on healthcare (GH principle 5 / ACP position 1)

##### Observations

Several positive initiatives in the past 4 years at SYP have focused on more effective provision of health services. Among them, the institution of medical records utilized by the traveling teams in the spoke communities realizes for the first time a basis for continuity in the primary management of chronic disorders. Balancing the work schedule of the permanent Guatemalan physicians between the hub clinics and traveling teams has engineered a horizontal care structure where none has previously existed. The efficiencies captured with a digitized pharmacy inventory and ordering system enhances the capability of the traveling teams and minimizes loss. These functionalities have virtually eliminated the need for traveling teams to carry bulk pharmaceuticals or leave behind unused agents that could be dispensed inappropriately. Controlling the number of villages in the scope of service commensurate with the capacity of staff and resources is essential to the success of the model. These incremental measures have been the result of a board and medical committee deliberating specifically on healthcare within this set of communities.

Board and medical committee members are now considering mechanisms for networking with other NGOs operating in the region that provide specialized services such as prenatal and early childcare instruction, as well as the best way to reconstitute the Amigas role and function.

It may be a current advantage of omission that SYP is not involved in educational programs of university students or health professionals. Many of the pitfalls of concern in global health volunteering by students, medical or surgical residents center on inadequate preparation, inappropriate practice by trainees on vulnerable host populations, and the investment exchange motivation related to enhancing ones curriculum vitae over the objective of providing public goods [10, 11]. Understanding how these conflicts arise and coordinating proactively with educators in both countries, on the other hand, could open allopathic and osteopathic medical colleges to the client portfolio of SYP at the proper time in its development, particularly if coordinated with a host country medical college.

##### Recommendations

SYP should court medical, surgical, and dental team sources that lack a host partner that is as well-organized. SYP should also consider publishing its developmental experiences and model as well as sharing information on its de novo software for pharmacy ordering and inventory that can be implemented on a small scale and budget.

The contracted physicians have rendered a prioritized wish list of portable diagnostics to expand the primary care services and chronic care management of the spoke communities. It is recommended that SYP explore the availability of ruggedized, low maintenance technology solutions to for these objectives.

The notice taken of how mobile telephony has enhanced the ability to effect continuity of care in this environment is a bellwether of possibilities. Though WIFI or other private internet access is unavailable, some villages may soon have data signal reach. Depending on the prevalence of smart phones, educational health messaging to entire communities could be made available in their own dialect. Announcing regularly when the next team is expected in villages may serve to keep health practices upfront in the minds of villagers, promote responsible health dialogue, personal and community support systems, and peer-to-peer awareness. Telemedicine links with remote volunteer specialists could follow. The Guatemalan staff of SYP could take the lead and coordinate the effort with the Amigas initiative for local optimization. The leap-frog advances afforded by smart phone proliferation in India exemplify the disruptive benefits to education and healthcare for the poor that could be replicated in the foreseeable future in Guatemala [12].

Each of these technical and workflow innovations improves quality and capacity in local services. Minutes of the board and medical committee suggests an effective review and action process that should be maintained.

It is paramount that the primacy of improving patient care not be subjugated to the educational objectives of the Global North. However, should a collaboration between host and northern medical universities develop, then membership in the Consortium of Universities for Global Health (CUGH) could serve as a source for innovative ideas to advance the health objectives of SYP and a strategy for capacity-building.

#### Mutuality of learning and respect for local health professionals and their essential contributions (GH principle 6 / ACP position 3)

##### Observations

No dispute exists among Guatemalan and US colleagues that there is neither displacement of nor competition with local private or public health personnel and clinics from the activities of SYP and its client visiting teams. Such resources simply are not present in the serviced communities due to lack of purchasing power. Reliance on contracted Guatemalan physicians in all core activities, protocols, and the medical committee anchors the NGOs in their common quest. Host and visiting physicians both acknowledge the satisfaction of interaction and would like to build on the dynamic.

Both the Guatemalan contracted physicians and visiting physicians from the US concur that the interaction of physicians is fruitful. An example from the current PVUMC trip is a US physician transferring to a Guatemalan physician the technique of ear canal lavage for cerumen impaction, and Guatemalan physicians refreshing US physicians on the diagnostic probabilities along the range of intestinal infections and application of oral rehydration salts. Contracted physicians provided a list of diagnostic studies in order of preference that would be desirable for the teams to be able to bring to the spoke communities to assess for and manage an increasing number of primarily chronic disorders.

##### Recommendations

Adopt the contracted physicians’ request that, going forward, all the physicians should meet on Sunday before a traveling team excursion takes place to review together expected common complaints and symptoms, protocols for managing common conditions, processes for dispensation of medication and protocols for arranging follow up.

## Conclusion

The standards published by GH and the ACP, in this case, served as a useful lens to assess SYP. As a result of the administrative consultation utilizing the standards, SYP accepts resultant recommendations as appropriate to their mission in a global context and plans to implement them when and where feasible. Further, SYP intends to adopt these standards and promote its adherence to them in fund-raising measures and in attracting client teams from additional high-income country NGOs that can demonstrate that their practices also conform.

The composite progress made by SYP in each of the standards’ dimensions allows US physicians to meet their ethical obligations while participating in STGHCE under the auspices of SYP. Expanding the depth of services for these host communities hinges on the availability of visiting physicians and support staff to meet the desired periodicity in the near and intermediate term.

The published standards used herein as benchmarks of performance should be applied in context rather than as absolutes. For example, respect for governance may be ambiguous where local inhabitants and health care providers fear government and resist attempts at universal identification schemes meant to augment patient follow-up. Registering traveling physicians with the host-country MoH may be ideal, yet a mechanism for doing so may be unavailable. Visiting physicians may also risk sacrificing local trust if seen to be too collaborative with public officials. Until local medical graduates can make a living in the service of their poorer compatriots, outside pro-bono assistance answers an unmet need.

Neither set of standards (GH or ACP) lists capacity-building within their respective “Principles” or “Positions” [1, 2]. Nonetheless, the published manuscripts do integrate capacity-building into the concept of sustainability. While global health principles impel that organizations like SYP should pursue their own obsolescence through building host capacity, their existence in real time rests on current external economic realities and a pace of progress not directly controlled by such organizations. This does not imply complacency nor an intention of permanency. Successful capacity-building, particularly in primary care, may require staging, patience, technology adoption, and assisting in efforts that ultimately empower remote poorer communities to capacitate the financial requirements of host medical manpower for which these communities must compete. This requires sustainability for the medium term, as emphasized in the standards. Further, SYP’s nascent investment in human capital within its elementary school is a seed for that development and could eventually become a local draw for the children of the host physicians that these communities vitally need.

Skill in the application of any new tool should improve with experience. Missed opportunities and shortfalls in the conduct of this inaugural application of the standards in real-life administrative consultation may be many and identified in retrospect. Are the content and mode of delivery of the preparatory programs of SYP and PVUMC what they should be? How is review of those processes undertaken? How does an organization like SYP forecast, track, and influence the drivers of regional economic development essential to remediating health inequities?

SWOT analysis has the advantage of inherent simplicity in organizing responses to exploratory questions in multi-source/multi-rater feedback (360-degree) universes. That simplicity may be a disadvantage in complex environments with major elements that the organization does not control. Using a 360-degree approach concomitantly optimizes the opportunity for triangulating among sources for verification and for grounded exploration, but can be quite time-consuming and tedious. Other qualitative and quantitative methodologies may serve better when the right questions become clearer. The advantage of a consultative approach as used in the administrative report to SYP is that it allows the busy staff of a budget-constrained health NGO to obtain a dispassionate third-party evaluation, and to reorient their compass using consensus wisdom largely devoid of unavoidable personal agendas. The disadvantage is that an operational assessment cannot measure changes in community health status, local economy, and quality of life in the Global South occurring as a result of STGHCE from the Global North. The lack of tools in that dimension remains the key weakness related to this form of transnational interaction. NGOs like SYP should remain vigilant of the social science as it develops in this arena and share their own innovations as they arise. Studies using matched control communities with carefully selected variables could be of value in developing best practices to enhance universal horizontal primary care enabled through STGHCE.
